# miR451 and AMPK Mutual Antagonism in Glioma Cell Migration and Proliferation: A Mathematical Model

**DOI:** 10.1371/journal.pone.0028293

**Published:** 2011-12-20

**Authors:** Yangjin Kim, Soyeon Roh, Sean Lawler, Avner Friedman

**Affiliations:** 1 Department of Mathematics and Statistics, University of Michigan, Dearborn, Michigan, United States of America; 2 Department of Natural Sciences, University of Michigan, Dearborn, Michigan, United States of America; 3 Translational Neuro-Oncology Group, Leeds Institute of Molecular Medicine, University of Leeds, Leeds, United Kingdom; 4 Mathematical Biosciences Institute, Ohio State University, Columbus, Ohio, United States of America; University of Michigan School of Medicine, United States of America

## Abstract

Glioblastoma multiforme (GBM) is the most common and the most aggressive type of brain cancer; the median survival time from the time of diagnosis is approximately one year. GBM is characterized by the hallmarks of rapid proliferation and aggressive invasion. miR-451 is known to play a key role in glioblastoma by modulating the balance of active proliferation and invasion in response to metabolic stress in the microenvironment. The present paper develops a mathematical model of GBM evolution which focuses on the relative balance of growth and invasion. In the present work we represent the miR-451/AMPK pathway by a simple model and show how the effects of glucose on cells need to be “refined” by taking into account the recent history of glucose variations. The simulations show how variations in glucose significantly affect the level of miR-451 and, in turn, cell migration. The model predicts that oscillations in the levels of glucose increase the growth of the primary tumor. The model also suggests that drugs which upregulate miR-451, or block other components of the CAB39/AMPK pathway, will slow down glioma cell migration. The model provides an explanation for the growth-invasion cycling patterns of glioma cells in response to high/low glucose uptake in microenvironment *in vitro*, and suggests new targets for drugs, associated with miR-451 upregulation.

## Introduction

**Figure 1:**
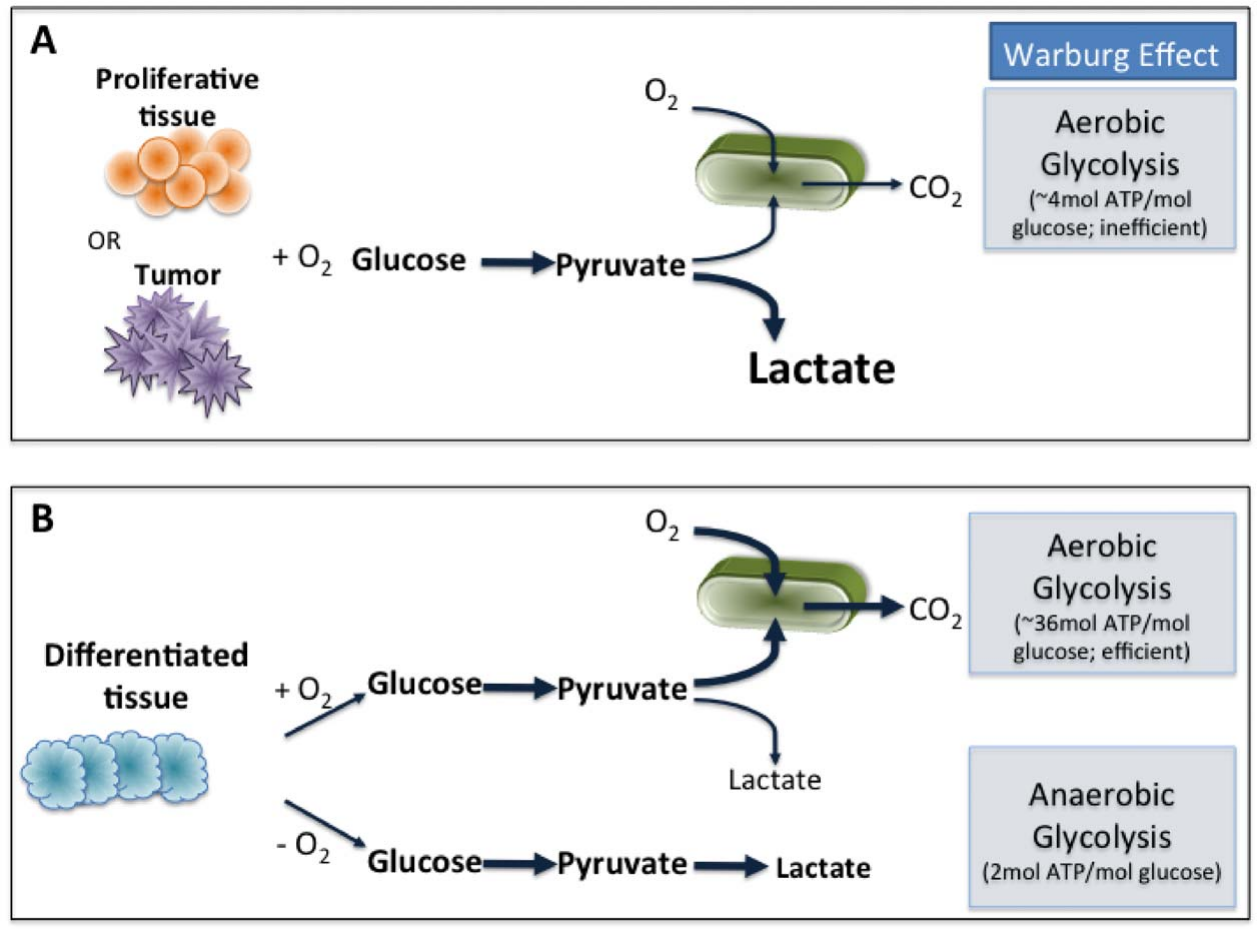
Schematics of oxidative phosphorylation, anaerobic glycolysis, and aerobic glycolysis. (A) To enable rapid proliferation, cancer cells shift their metabolic machinary toward high levels of glucose uptake and lactate production (aerobic glycolysis, the Warburg Effect”). (B) Differentiated cells favor oxidative phosphorylation and anaerobic glycolysis.

Glioblastoma is the most common and aggressive brain tumour, marked by high levels of proliferation angiogenesis and invasion [1]. These tumors are highly invasive and the median survival time from the time of diagnosis is approximately one year. Tumor cells may face hypoxia, acidity, and limited nutrient availability as they grow. In order to maintain rapid growth, glioblastoma cells must remain highly adaptive in response to changes in the microenvironment [2]. To enable rapid proliferation, cancer cells shift their metabolic machinary toward high levels of glucose uptake and lactate production (the “Warburg Effect”) [3], [4]. While differentiated cells favor oxidative phosphorylation and anaerobic glycolysis, proliferative tumor cells adapt what appears to be a less effective way of metabolism, *i.e.*, aerobic glycolysis [5]; see Figure 1. While the tricarboxylic acid (TCA), or Krebs, cycle (schematically described by the pathway in Figure 1) is a key step for generating ATP in nonhypoxic normal cells, cancerous cells consume large amounts of glucose and generate lactic acid rather than using the TCA cycle [4]. Cancer cells ensure an adequate glucose supply through increased angiogenesis and migration [2] and cellular responses to glucose withdrawal may be critical for the survival of cancer cells in rapidly growing tumors such as glioblastoma, where glucose levels may fluctuate. Cancer cells therefore engage strategies of metabolic adaptation to survive periods of metabolic stress and maintain viability as cells accumulate [6]. The 5′-adenosine monophosphate activated protein kinase (AMPK) pathway is the major cellular sensor of energy availability [7]. AMPK is a conserved cellular energy sensor that is activated by metabolic stress to promote energy conservation and glucose uptake [7]. This allows cells to adapt to periods of low energy availability, thus avoiding bioenergetic catastrophe and cell death.

**Figure 2:**
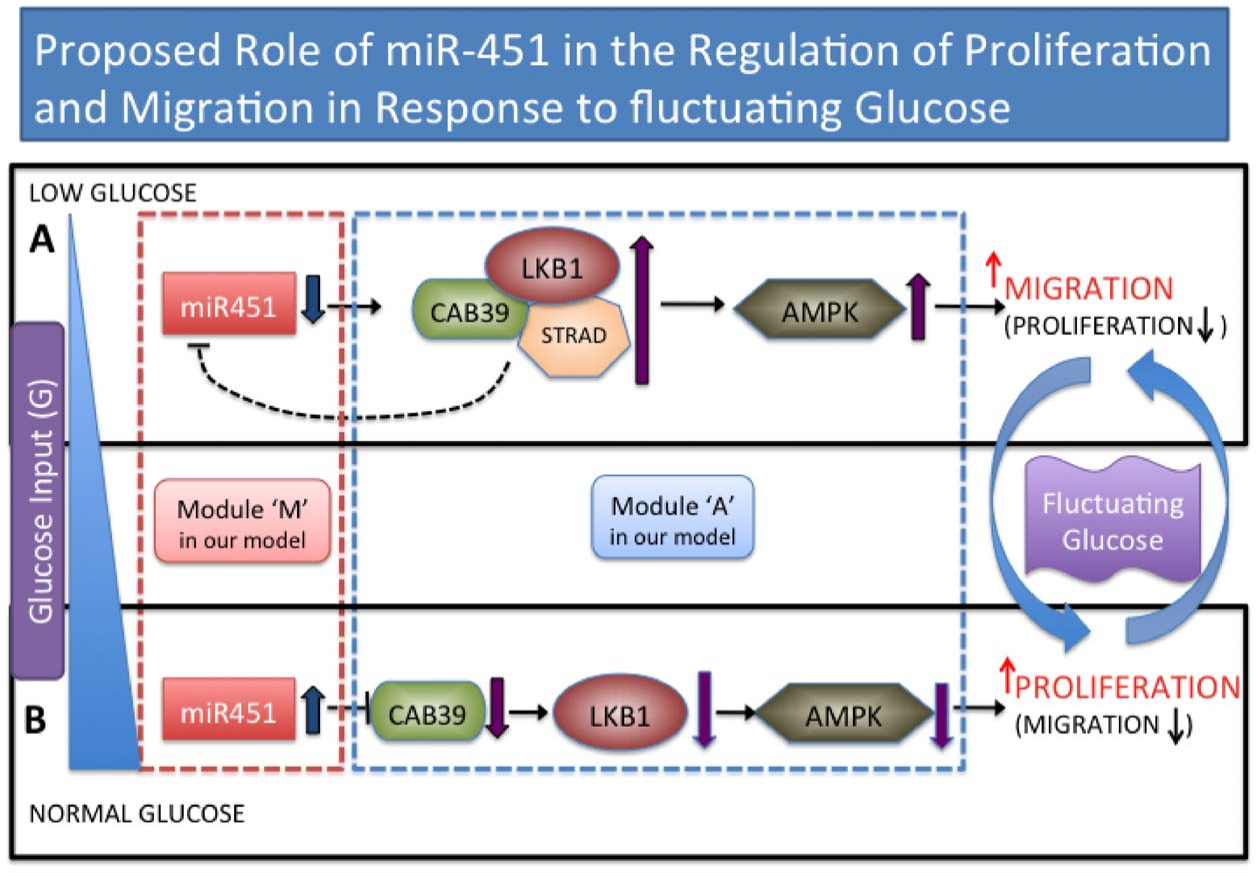
Proposed role of miR-451 in the regulation of LKB1 signaling in response to fluctuating glucose. miR-451 levels determine cell migration or proliferation in response to glucose (blue triangle on the left). (A) Low glucose level reduces miR-451 and leads to upregulation of AMPK and enhanced cell motility. (B) Normal glucose level upregulates miR-451, which in turn leads to increased proliferation and decreased cell migration by inhibiting CAB39-LKB1-AMPK pathway. Schematic components of miR-451 and the CAB39/LKB1/AMPK complex is represented by modules ‘M’ (box with red dotted line) and ‘A’ (box with blue dotted line) in our theoretical framework. Blue arrows on the right indicate the switching behavior between a migratory mode in (A) and the proliferative state in (B) in response to fluctuating glucose.

In addition to rapid proliferation, invasion of glioma cells from the core tumor into the surrounding brain tissue is a major reason for treatment failure: the migrating cells are not eliminated in surgical resection and promote tumor recurrence. Godlewski *et al.* [2] have recently identified a novel mechanism in which glioma cell survival, motility and proliferation are coordinately regulated by a single microRNA (miR-451) that regulates AMPK signaling in response to glucose levels in glioblastoma cells. Their model is shown schematically in Figure 2. In this study, miR-451 was shown to play a key role in glioblastoma by modulating the balance of active proliferation and invasion in response to metabolic stress in the microenvironment. Normal glucose levels allow high miR-451 which in turn leads to elevated proliferation and decreased cell migration, whereas the low glucose level downregulates miR-451 thereby leading to enhanced cell motility and migration.

Kim *et al.* [8] recently developed a mathematical model of invasive glioma cells in 3D tumor spheroids. The model was shown to be capable of reproducing migration patterns of glioma cells in *in vitro* experiments, exhibiting, in particular, dispersion and branching of cells. The model included MMP activity and glucose levels as well as chemotaxis, haptotaxis and cell-cell adhesion forces. The rapid migration of cells is caused primarily by the chemotaxis forces that are associated with glucose concentration 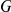.

In the present paper we explore in more detail the effect of glucose on glioma cell behavior with the aim of suggesting drug targets that will slow cell migration. In the present work we represent the miR-451/AMPK pathway by a simple model and show how the effects of glucose on cells need to be “refined” by taking into account the recent history of glucose variations. We simulate the model of Kim *et al.* [8], taking the glucose input to vary periodically. The simulations show how variations in glucose significantly affect the level of miR-451 and, in turn, cell migration. Importantly, the model predicts that oscillations in the levels of glucose increase the growth of the primary tumor. The model also suggests that drugs which upregulate miR-451, or block other components of the CAB39/AMPK pathway, will slow down glioma cell migration.

## Materials and Methods

### Mathematical modeling of miR-451-AMPK control

**Figure 3:**
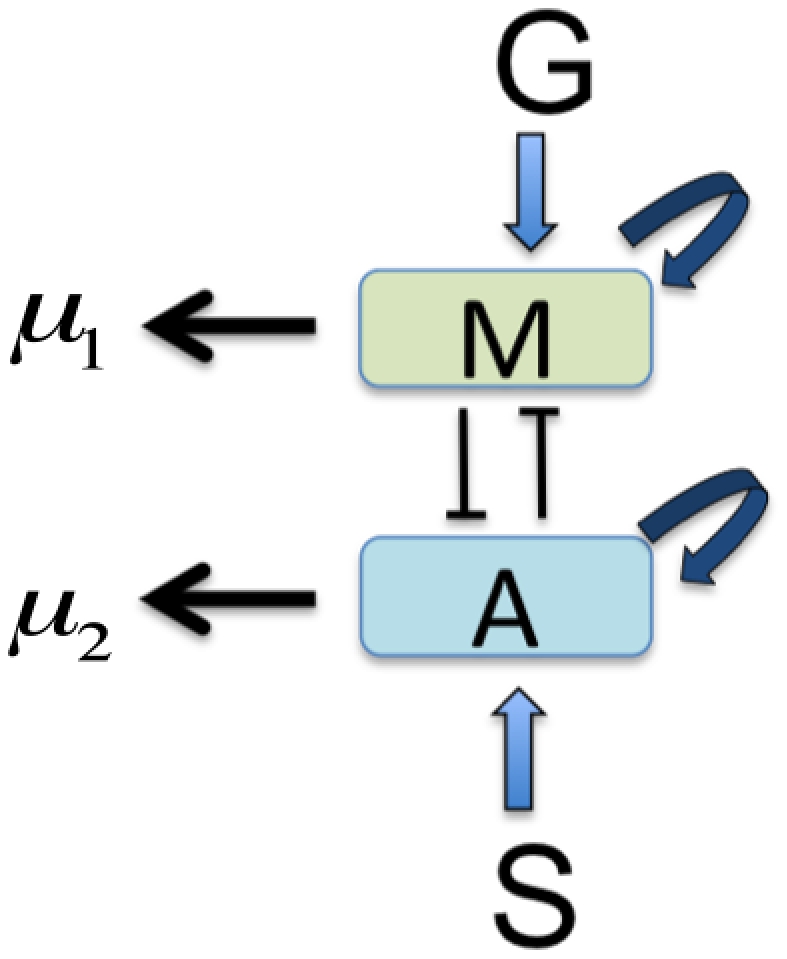
A simplified model of the network shown in Figure 2. Two key molecules, miR-451 and AMPK complex, are represented by 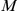 and 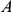 respectively. The dynamics of miR451 includes input from glucose (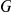), decay (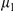), and autocatalytic increase. AMPK complex is induced by source (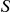), and undergoes autocatalytic increase with natural decay (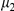). In addition, there are mutual inhibition mechanisms between 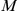 and 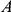.

In order to incorporate the signaling network shown in Figure 2 into our model of glioma cell migration, we began by simplifying this network. We represent by 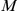 and 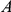 the activities of miR-451 and AMPK complex, respectively. We also denote the signaling pathways to miR-451 and AMPK complex, by 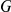 (glucose) and 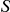 respectively. Figure 3, with protein degradation at rates 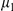 and 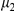 (for 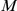 and 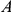), is a simplified representation of Figure 2. The scheme includes autocatalytic activities of 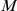 and 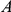 as reported in [2] and the inhibiting signal suggested in Figure 2A.

To mathematically model the dynamic network shown in Figure 3 we introduce the following variables:

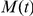
 = concentration of miR-451 at time 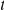,

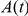
 = concentration of the complex CAB39/LKB1/AMPK at time 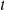,

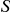
 = source of the complex 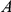,

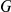
 = glucose input level,

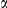
 = inhibition strength of miR-451 by the complex,

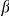
 = inhibition strength of the complex 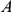 by miR-451,

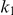
 = autocatalytic enhancement rate of miR-451

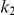
 = autocatalytic enhancement rate of the complex.

We associate to the network in Figure 3 the dynamical system, written in dimensionless form,
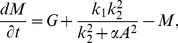
(1)

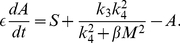
(2)
Table 1 summarizes the parameters used in equations (1)–(2), and some explanations are given in the Text S1. The strength of the inhibition of miR-451 by the complex 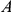 can be controlled through the dimensionless parameter 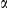, and the strength of the inhibition of the complex 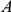 by miR-451 can be controlled through the dimensionless parameter 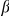. The parameter 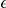 is given by 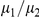, the ratio of the degradation rates of 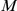 and 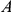, respectively. Since miR-451 degrades much faster than the complex 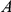, 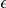 is a very small number (we shall take 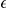 = 0.02). We recall (see Figure 2) that elevated levels of miR-451 imply elevated cell proliferation and reduced migration, whereas low levels of miR-451 imply reduced cell proliferation and increased migration. In order to incorporate the effect of glucose into our model of glioma cell migration, we need to determine how the level of glucose 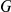 affects the level of 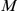.

**Table 1:** Parameters that are used in the internal dynamics model (miR-451/AMPK system).

	Description	Value	Refs
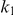	miR451 autocatalytic production rate	4.0	TW
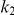	Inhibition parameter of miR-451 by AMPK complex	1.0	TW
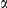	Inhibition strength of miR-451 by AMPK complex	1.6	TW
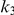	AMPK autocatalytic production rate	4.0	TW
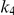	Inhibition parameter of AMPK complex by miR-451	1.0	TW
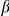	Inhibition strength of AMPK complex by miR-451	1.0	TW
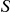	Signaling source of AMPK complex	0.2	TW
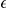	Scaling factor (slow dynamics)	0.02	[12]–[14],TW
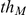	Threshold value of miR-451 for the invasion/growth switch	2.0	TW

TW = This work.

**Figure 4:**
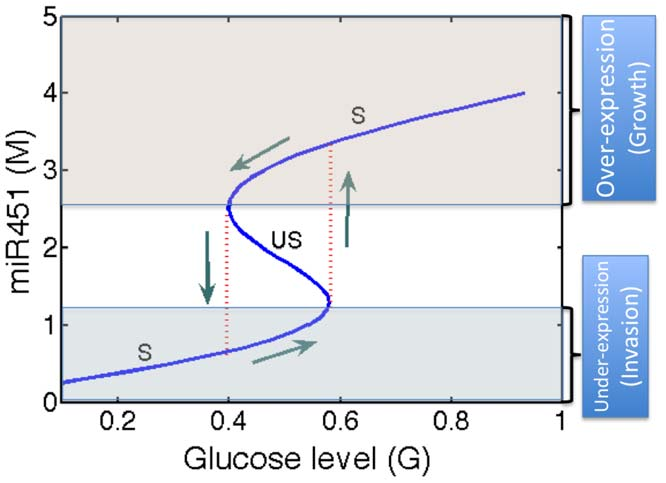
The 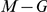 hysteresis loop: 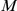 is upregulated when 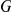 varies in the upper stable branch, and downregulated when 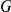 varies in the lower stable branch. We define the migration region by 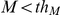 and the proliferation region by 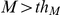, and take 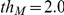.

When the main miR-451-AMPK system (1)–(2) is in equilibrium, we can solve miR-451 levels (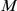) as a function of injected glucose amounts (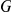) for any set of parameters 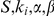. Figure 4 shows the graph 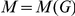 as 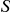-shaped curve when the parameter values are 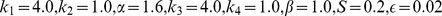. The upper and lower branches are stable, and the middle branch is unstable. If 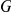 is small, then the system (1)–(2) is in the lower branch, 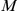 is low, and the glioma cells are in migratory phase. This situation continues to hold as 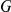 is increased until it reaches the value 0.6. At this point, the system jumps to the high branch, with an elevated level of miR-451, and the cells are in the proliferative phase (while migration is reduced). As 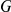 is decreased, the level of miR-451 remains elevated, until 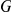 is decreased to the level 0.4, at which time miR-451 jumps to the lower branch, and the cells return to the migratory phase. We conclude that the effect of glucose is history dependent: when 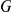 is at an intermediate level, 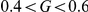, the cells are in the migratory phase if 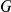 was in increasing mode, and in the proliferative phase if 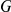 was in decreasing mode.

Figure 4 suggests that a state (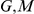) with 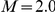 will be moved by the dynamical system (1)–(2) into the upper stable steady state branch, resulting in overexpression of miR-451 and, thus, in growth. On the other hand, if 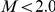, then (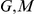) will end up in the lower stable state branch, which implies migration. Hence we shall define the migratory region by 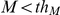 and the proliferation/growth region by 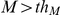 and take the threshold 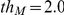.

**Figure 5:**
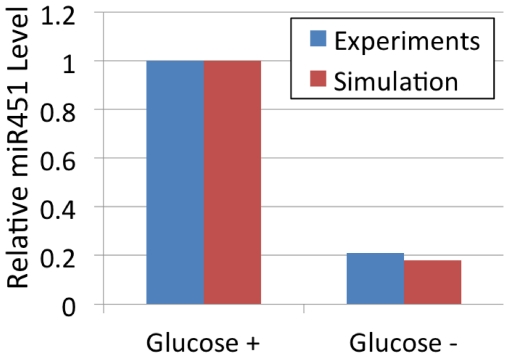
Experiments vs. simulation. Effect of glucose deprivation on miR-451 expression levels. In [2], miR-451 level was reduced by 21% for different cell lines (U251, LN229) after cells are cultured for 24 hr in normal (+, glucose 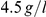 or reduced glucose (−, glucose 0.3 g/l). Simulation results (red) are in good agreement with experimental results (blue; U251 cell line) from [2]. Y-axis in the figure is the relative miR-451 concentration for high (glucose+; dimensionless value 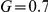 in simulation) and low (glucose−, glucose−, 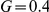 in simulation) level of glucose in the medium.

In the next section we shall incorporate this phenomenon into the model which was developed in Kim *et al.* [8]. We conclude this section by comparing predictions of the model (1)–(2) with experimental results of Godlewski *et al.* [2]. In the experiments, the miR-451 level was reduced by 80% when cells were cultured in low glucose (0.3 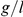) compared normal glucose (4.5 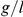). Our simulation results, shown in Figure 5, are in good agreement with these experimental results. Indeed, with the parameter set as in Figure 4, the model predicts slightly more than 80% reduction of miR-451 levels in low glucose.

### Mathematical model of the complete dynamics

In [8] we previously developed a mathematical model of migration patterns of glioma cells. The model included the forces of cell-cell adhesion, haptotaxis, chemotaxis induced by a glucose gradient, and shedding of glioma cells from a spheroid glioma tumor. In the present paper our aim is to examine in more detail the effect of glucose on cell migration. In order to elucidate the dependence of glioma cell migration on the gradient of glucose 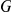, we shall vary 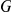 in a periodic manner at a location away from the primary tumor and determine how these changes affect the proliferative and migratory phases of the cells. In doing so we may ignore the shedding of cells, as this will not affect how fast cells migrate after they leave the primary tumor. We may also ignore the cell-cell adhesion, since this force only affects the clumping pattern of cells, not their proliferation rate or speed of migration. Thus in order to describe the effect of 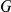-oscillation on migratory/proliferative phases of glioma cells, we ignore shedding and cell-cell adhesion, but include haptotaxis and chemotaxis. On the other hand we shall incorporate the observation (modeled by (1)–(2)) that tumor cells react to miR-451 expression in response to the glucose level.

**Figure 6:**
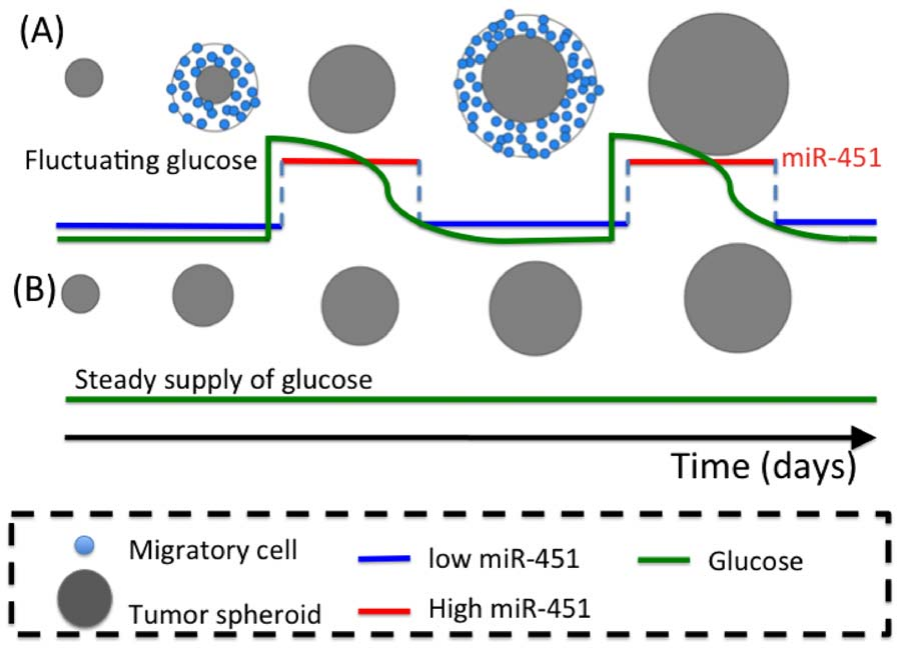
Two different growth schemes of tumor spheroids (gray filled circle) with same initial size in response to fluctuating and steady glucose (green solid line). (A) Cycle of growth and invasion in response to fluctuating glucose. Tumor cells (blue filled circle) at the surface of the spheroid begin to migrate away from the core when miR-451 levels are low (blue solid line; high AMPK activity) due to low glucose. These migratory cells become proliferative cells when high glucose is introduced and miR-451 levels are high (red solid line; low AMPK activity). (B) Monotonic growth due to steady supply of glucose; the total supply of glucose is the same as in (A). We hypothesize that tumor spheroids with fluctuating glucose in (A) will grow faster than ones grown in steady glucose supply in (B).

The model simulation will show that the effect of glucose oscillations is schematically captured by Figure 6: The same total amount of glucose, if injected in a fluctuating fashion, would lead to increased tumor growth.

### Tumor cell density 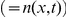


As noted in the previous Section, tumor cells begin to invade the surrounding environment when miR-451 levels are low and AMPK activity is high, and they proliferate when miR-451 levels are high and AMPK activity is low. This alternating behavior between migration and proliferation under the control of miR-451 is modeled using a threshold of miR-451 level, 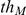, by the following equation
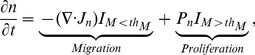
(3)where 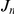 is the flux of the cells under the migration mode, 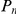 is the proliferation term under the growth mode, and 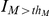 and 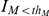 are indicator functions. They are discontinuous but made smooth in simulations.

The flux for movement consists of three components: random motility (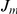), chemotaxis (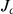), and haptotaxis (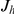).
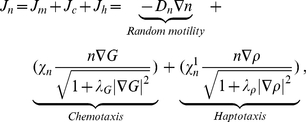
(4)where 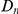 is the random motility, 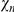 is the chemotactic sensitivity, 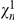 is the haptotactic coefficient, and 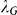 and 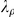 are chemotactic and haptotactic parameters respectively. 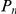 represents a logistic growth under proliferation mode:
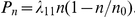



### ECM 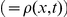


ECM is degraded by MMP and undergoes remodeling/reconstruction,
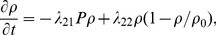
(5)where 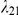 is the ECM degradation rate by MMP, 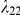 is the reconstruction parameter, and 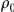 is the ECM carrying capacity.

### MMP 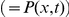


MMP is produced by invasive tumor cells in the presence of the ECM and is usually localized near the invading cell front (small diffusion),
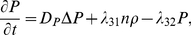
(6)where 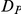 is the diffusion coefficient, 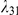 is MMP production by tumor cells (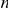), and 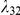 is the natural decay rate.

### Glucose (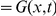)

To define the oscillation of the glucose supply, we denote by 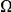 a region which includes all the glioma cells, and by 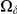 a subregion which lies far away from the initial location of the glioma cells. We choose discrete times 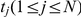 with a period of 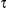 (that is, 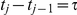 for all 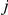) and introduce the indicator functions
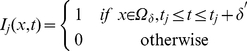
(7)where 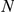 is the number of injections that take place during intervals 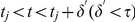. We assume that the concentration of glucose (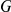) satisfies a reaction-diffusion equation on the whole domain 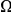:
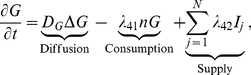
(8)where 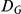 is the diffusion coefficient, 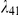 is the consumption rate of glucose by tumor cells, and the last term is the glucose supply rate from the far away field.

### miR-451 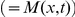 and AMPK 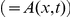


The two internal key variables 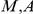 (miR-451 and AMPK) for the control of growth and invasion at each tumor cell are linked to tumor cell density above (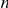):
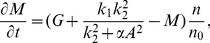
(9)

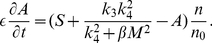
(10)


### Governing equations

Collecting the equations derived above for the tumor density (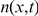), concentrations of the ECM (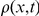), MMP (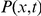), glucose (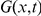), miR-451 (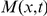), and AMPK (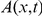), we have
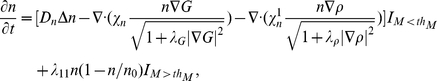
(11)

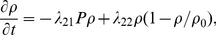
(12)

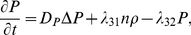
(13)

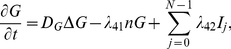
(14)

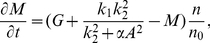
(15)

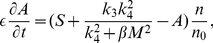
(16)where 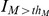 is the indicator function of growth region (where miR-451 level (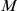) is greater than a threshold value (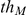)) and 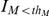 is the indicator function of the invasive region (where miR-451 level is below the threshold (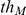) ).

The parameter values and the reference values for equations (11)–(14) are given in Tables 2 and 3, respectively. Some of the parameters are taken from the literature and others are estimated. Text S1 explains the choice of the parameters and reference values, and the non-dimensionalization scheme.

**Table 2:** Parameters used in the tumor model.

	Description	Dimensional value	Dimensionless	Refs
Diffusion coefficients				
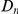	tumor cell in spherical core	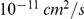	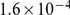	[8]
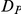	MMPs	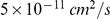	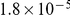	[8], [15], [16]
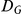	Glucose	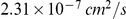	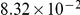	[8], [17],TW
Production/consumption rates				
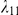	tumor cell growth rate	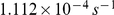	0.1	[18], [19], TW
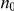	carrying capacity of tumor cells	= 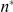	1.0	TW
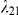	ECM degradation rate	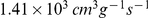	0.508	[8],TW
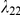	ECM release/reconstruction rate	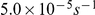	0.18	[8],TW
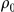	ECM carrying capacity	= 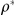	1.0	[8]
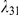	MMP producdtion rate	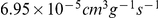	2.5	TW
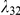	MMP decay rate	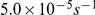	0.18	[8],TW
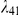	glucose consumption rate	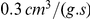 .	1.0	[19], [20], TW
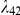	glucose injection rate	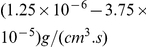	1–30	TW
Movement parameters (chemotaxis, haptotaxis)				
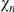	chemotactic sensitivity parameter	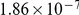 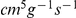	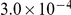	TW
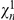	haptotactic parameter	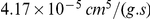	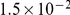	[8],TW

**Table 3:** Reference variables used in the model.

Var	Description	Value	Refs.
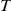	Time	1 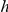	
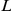	Length	1.0 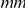	
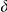	glucose injection domain	0.08 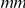	TW
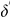	injection duration	0.01 h	TW
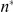	tumor cell density	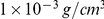	TW
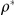	ECM density	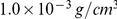	[8], [21], [22]
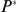	MMP concentration	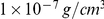	[8], [23]
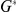	Glucose concentration	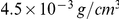	[2], [19], [24]

All the simulations in the Results section were performed using a finite volume method and clawpack (http://www.amath.washington.edu/~claw/) with a fractional step method [9] as well as the non-linear solver *nksol* for algebraic systems. Equations (11)–(16) were solved on a regular uniform spatial grid (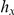 = 0.01). An initial time step of 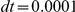 was used, but adaptive time stepping based on the number of iterations did increase or decrease this step size.

## Results

### Key control system : miR-451-AMPK network

Consider a spherical brain tissue, 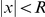, with glioblastoma tumor occupying a sphere 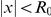 and glucose source at 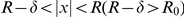. Glucose is consumed by tumor cells, resulting in low glucose concentrations near 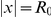 and relatively high glucose concentrations near the far field 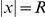. This creates a gradient field of glucose. Under this microenvironmental condition, the glioblastoma cells tend to migrate toward the glucose rich region, *i.e.*, towards the far field, 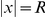. Indeed, glioblastoma cells are known for their particular tendency to metabolize glucose, through aerobic glycolysis, called the Warburg effect; recall Figure 1. Furthermore, the cells in the tumor core, starving and accumulating toxic waste materials, are sending ‘escape’ messages through hand-hand signaling toward the cells at the surface 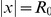 of the tumor, further encouraging them to invade into the far field. In our model low levels of miR-451 (high level of AMPK activity) due to low glucose levels at cell sites trigger tumor cells to initiate invasion toward 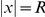, and keep invading until the miR-451 level creeps above a threshold (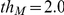) (or AMPK activity level drops below a threshold (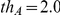)). For simplicity we carry out the simulations of the model equations (11)–(16) in the one-dimensional case. The computational domain is 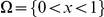, and we take 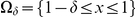. The glioma cells begin to migrate into 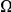 from the end-point 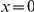. Glucose is consumed by tumor cells initially on the left side of the domain leading to low glucose concentrations near 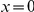 and relatively high glucose concentrations in the far field (near 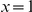).

### Simulation results

**Figure 7:**
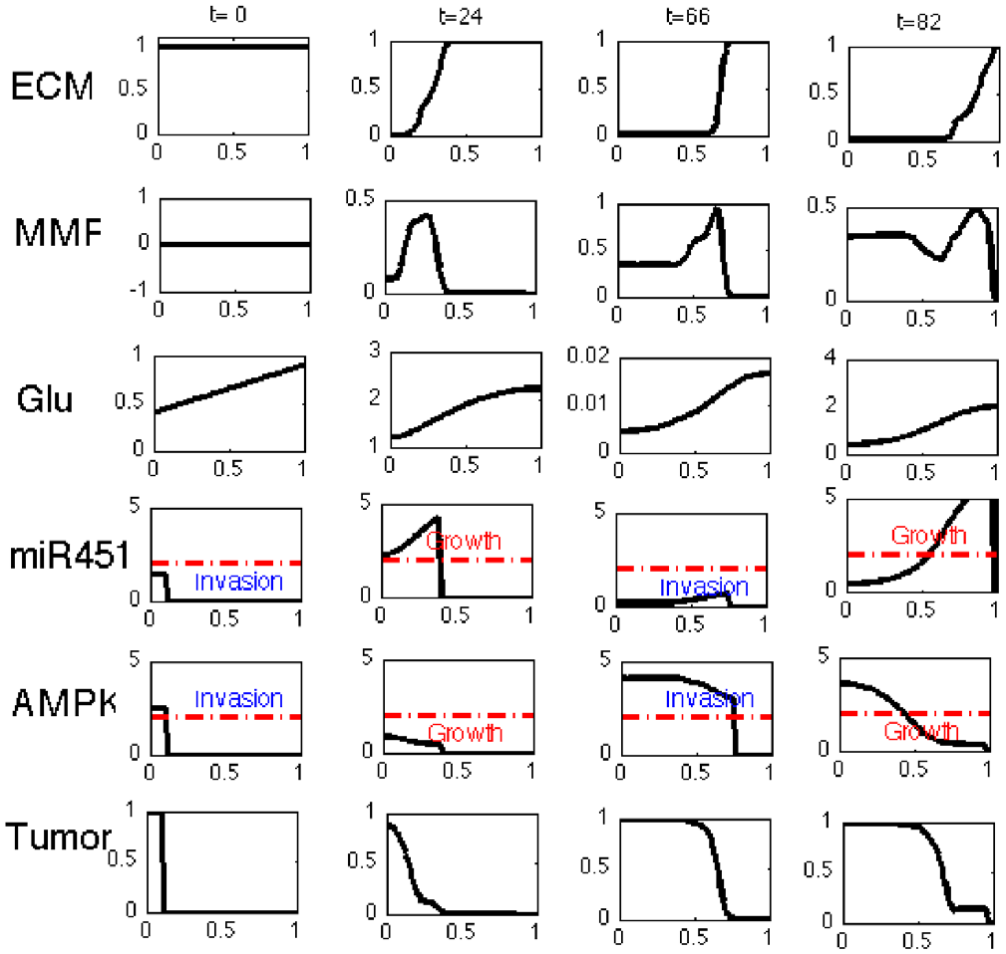
Typical evolution of spatial profiles of tumor density (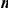) and concentrations of ECM (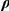), MMPs (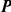), glucose (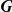), miR-451 (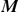), and AMPK (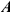). In each subfigure x-axis indicates the computational domain, left end (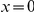) being the center of tumor mass and right end (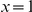) being the farthest field away from the tumor. Glucose was provided on the right hand side of the domain near 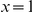 and glucose is transported by diffusion. Injection of glucose (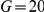) was given on the right side of domain (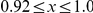) at time 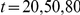. Levels of miR-451 and AMPK change in response to fluctuating glucose and determine whether cells would invade or grow. The red dotted line (−.) indicates the threshold 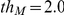.

Figure 7 shows a typical time course of tumor density (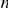) and concentrations of ECM (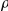), MMPs (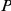), glucose (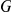), miR-451 (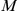), and AMPK (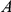) in response to a periodic injection of glucose into the system. Tumor cells were initially located on the left-hand side of the domain [0,1], near 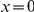. Glucose is consumed by tumor cells creating a gradient of glucose with higher levels at more distant areas. This lowered glucose level induces low miR-451 levels and high AMPK activity. Tumor cells near the surface of the tumor mass (with cell density 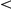
10%) begin to invade into the medium (toward the right) through chemotaxis (migration toward gradient of glucose) and haptotaxis (migration toward gradient of ECM using MMPs). MMPs are localized near moving front cells, and the high level of MMPs degrades ECM. This invasion continues until another flux of glucose is introduced to the system. Glucose injections at discrete times 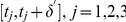 (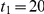, 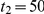, 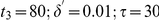), then induce a high level of miR-451 (low AMPK activity) enabling tumor cells in the invasive region to begin to grow again. One can observe fluctuating miR-451 levels and AMPK activities in response to glucose levels.

**Figure 8:**
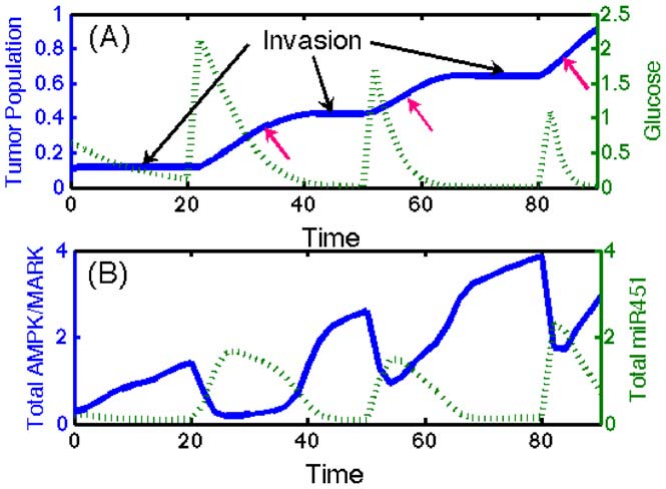
Dynamics of tumor invasion/growth. (A) Time evolution of total tumor population and total glucose level. Cells adapt growth phase (red arrows) via low AMPK level (high miR-451) when glucose supply is high and invasive phase (black arrows) via high AMPK level (low miR-451) in response to low glucose levels. (B) Time evolution of total concentrations of miR-451 and AMPK. High peaks of miR-451 are consistent with high peaks of glucose in (A) and lead to low levels of AMPK. *Data from Figure 7.

Figure 8 shows a time course of total tumor population and the levels of glucose, miR-451, and AMPK. Fluctuating glucose levels in (A) lead to a peak of miR-451 level and low AMPK activity. In turn, fluctuating AMPK levels give rise to plateau invasion mode (marked as black arrow in (A)) and creeping growth curve (red arrow in (A)) of the tumor population due to invading cells near the tumor surface when the AMPK level is high.

**Figure 9:**
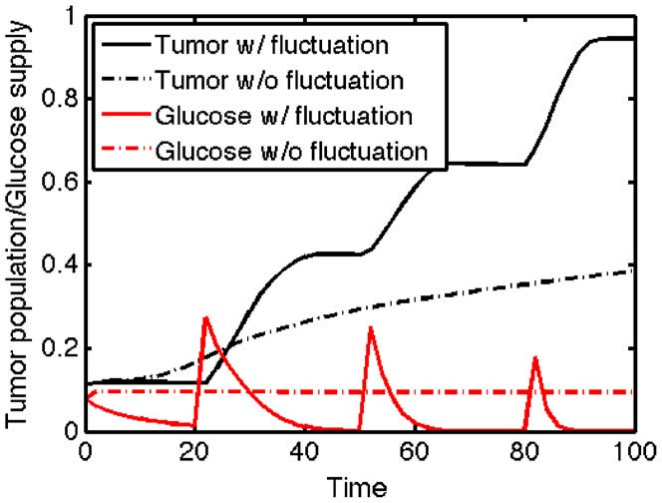
Comparison of tumor growth. Time evolution of total tumor population and total glucose level near far away field. In responds to periodic input of high glucose, tumor population shows alternating growth and invasion pattern (solid;black). When same amount of glucose without glucose fluctuation was provided to the system near 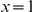, tumor population growth is induced from diffused glucose level and followed high miR-451 and low AMPK concentration at cell site. This induces a low chance of cell invsion near the surface and eventually total growth is low compared to the case with fluctuating glucose level.

Figure 9 shows that when the total supply of glucose is fixed the growth rate of the tumor with glucose fluctuation (black solid line) is larger than one with fixed supply of glucose (dotted black line).

**Figure 10:**
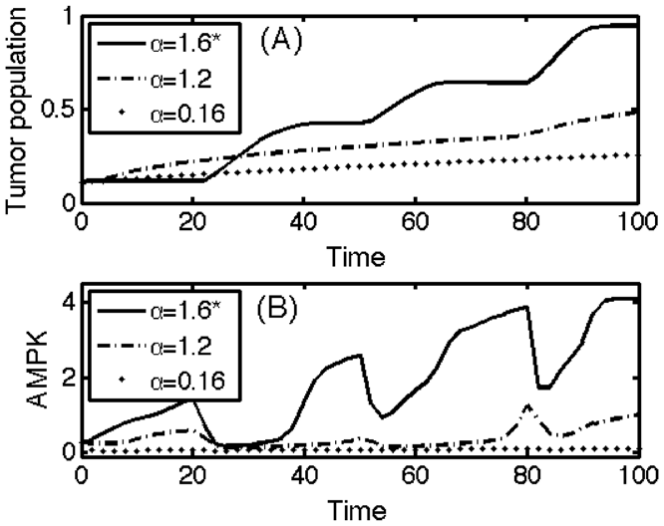
Effect of inhibition strength (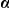) of miR-451 by AMPK. (A–B) Time evolution of tumor population and AMPK concentration within the system for different values of 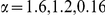. The control case was marked as star (*) for each subfigure.

In Figure 10, we show the effect of inhibition strength (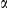) of miR-451 by the AMPK complex (more specifically, by the LKB1/STRAD complex; see Figure 2A) on the tumor population. In contrast to the control case (marked as star (*)), the tumor population does not fluctuate (Figure 10A) and shows slower growth as 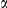 is decreased. Lower values of inhibition strength 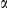 mean higher levels of miR-451 which induce low AMPK activity in Figure 10B. Blocking miR-451 along the pathways from CAB39/LKB1/STRAD/AMPK to miR-451 could be therefore a possible therapeutic target.

**Figure 11:**
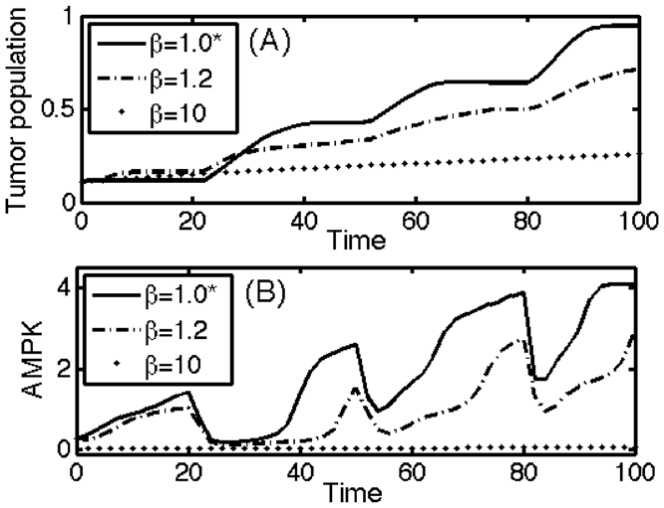
Effect of inhibition strength (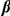) of AMPK by miR-451. (A–B) Time evolution of tumor population and AMPK concentration within the system for different values of 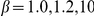. The control case was marked as star (*) for each subfigure.

In Figure 11, we show the effect of inhibition strength (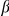) of AMPK complex by miR-451 on the tumor population. In contrast to the control case (marked as star (*)), the tumor population does not show invasion-growth patterns (Figure 11) for large 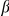 value and shows slower growth as 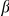 is increased. For intermediate values of 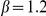, periodic fluctuation of AMPK is observed but the duration of high AMPK level is not as long as on the case of the control (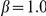). We also see, in the case of 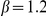, shorter invasion periods and slower growth than in the control case. Higher values of inhibition strength 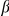 mean lower AMPK activity (in Figure 11B) which also induces high values of miR-451 and a much smaller tumor population. So, increasing inhibition of AMPK by miR-451 would have the same effect as decreasing inhibition of miR-451 by the AMPK pathway, and this suggests another possible therapeutic target.

**Figure 12:**
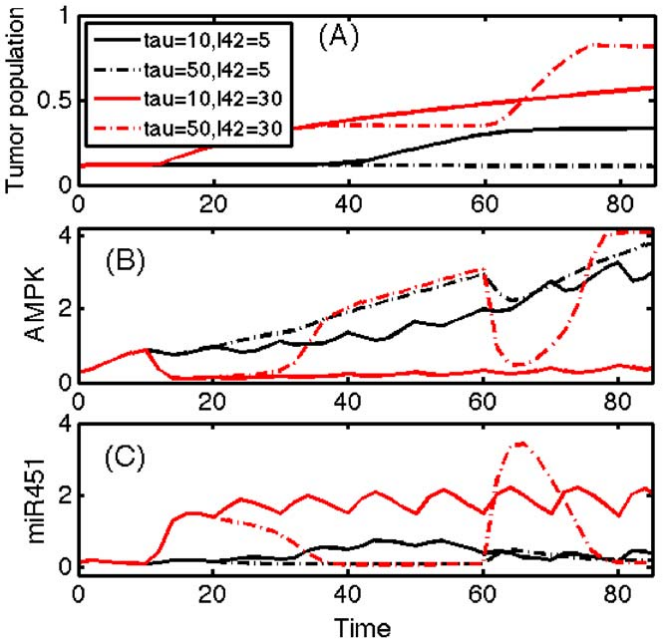
Time evolution of tumor population and the corresponding miR-451 and AMPK for different values of injection period (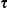) and injection supply amount (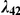).

In Figure 12, we show the time evolution of tumor population and total concentrations of AMPK and miR-451 for different values of injection period (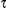) and injection amount (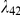). In the case of larger amounts of injection (red; 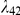 = 30), the overall tumor population grows faster than the smaller injection amount cases (black; 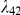 = 5). In the case of smaller amounts of injection, the low glucose level (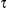 = 50) induces constant invasion without growth phase (black dotted line). In comparison, the frequent injection of glucose (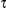 = 10) into the system creates a growth-invasion pattern. However, when the glucose is introduced into the system every ten hours (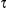 = 10), the system (of glucose and tumor cells) undergoes only three cycles of invasion and growth in 100 hours. This could be explained by the low miR-451 concentrations and high AMPK activity in Figure 12. In the case of larger amounts of glucose injected (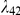 = 30), frequent injection leads to monotone growth phase while less frequent but appropriately small amounts of glucose injection lead to faster growth through cycles of invasion and growth. In the former case, as one can see in low AMPK concentration, tumor cells spend most of their time in growth phase due to abundant glucose with frequent large amounts of glucose injection. In the latter case, we also note that the number of the injections matches with the corresponding cycle, i.e. the system adapts to the growth-invasion phase each time glucose was administered. This dynamic behavior can also be confirmed in highly dynamic fluctuations in concentrations of miR-451 and AMPK.

## Discussion

Glioblastoma cancer cells shift their metabolic machinery toward a high level of glucose uptake, a phenomenon called the Warburg effect. Strong dependency of cells on glucose causes them to migrate in the direction of abundant glucose levels. Thus, the cancer cells migrate in the direction of increased glucose sources. Godlewski *et al.* [2] demonstrated that under low glucose levels glioma cells downregulate miR-451, and that this leads to upregulation of the AMPK kinases, which in turn increases the migration of cells while reducing their proliferation and growth. On the other hand, under normal (high) glucose levels, miR-451 is upregulated, leading to decreased AMPK activity, which results in elevated proliferation and growth but reduced migration.

In the present paper we developed a mathematical model of glioma cell migration and proliferation. In this paper, we considered the important role that miR-451 plays in glioblastoma. The model includes the concentrations of cancer cells, ECM, MMP, glucose, miR-451 and AMPK. Simulation of the model shows that by changing the level of glucose through periodic injections of glucose, the cancer cells will undergo nearly periodic changes from migration to proliferation modes. We showed how the effects of glucose on cells need to be “refined” by taking into account the recent history of glucose variations. The simulations show how variations in glucose significantly affect the level of miR-451 and, in turn, cell migration. The model predicts that oscillations in the levels of glucose increase the growth of the primary tumor. The model also suggests that drugs which upregulate miR-451, or block other components of the CAB39/AMPK pathway, will slow down glioma cell migration. In summary, the conclusions from the model include:

- Fluctuating glucose can lead to faster tumour growth and spread
- Response depends on the previous glucose levels, *i.e.*, whether it is rising or falling
- Targeting this pathway may reduce tumour growth by disrupting the cyclic responses to fluctuating glucose.

We suggest these simulation results as hypotheses to be tested experimentally. If the predictions of the model can be confirmed experimentally, we could proceed, side-by-side with the model and with experiments to determine how by modulating the injection of glucose, upregulating miR-451 (decreasing the 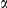 inhibition) and downregulating LKB1/STRAD (increasing the 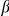 inhibition) we can decrease the invasion and the overall growth of the tumor. Simulations suggest that targeting this pathway may be beneficial in GBM treatments.

In this paper we used the concentration of tumor cells, not individual cells. Since even an isolated migrating glioblastoma cell may lead to faster spread and recurrence after surgery, it would be important to extend our paper by introducing cancer cells as individual agents. Hybrid approach will include cancer cells as individuals but the various chemical species as concentrations. A hybrid approach [10] has been proven to be important and useful in order to better understand the detailed mechanical interaction between a tumor cell and its microenvironment (see a recent review [11]). For simplicity we did not include in our model some important components of the microenvironment that may affect the growth and migration of glioma, notably fibroblasts, endothelial cells, and immune cells, as well as cytokines and growth factors secreted by these cells. There is currently only a limited understanding of the complex relationship between the tumor cells and the host cells in the microenvironment. A better understanding of this relationship may lead to new therapeutic approaches that target stromal elements instead of, or in addition to, tumor cells. We hope to address these situations in future work.

## Supporting Information

Text S1 **Detailed description of the models and parameters used.**
(PDF)
