# Supplementary material for: miR451 and AMPK Mutual Antagonism in Glioma Cell Migration and Proliferation: A Mathematical Model
Source: PLoS One. 2011 Dec 20;6(12):e28293. doi: 10.1371/journal.pone.0028293 (PMC3243681; doi:10.1371/journal.pone.0028293)
Supplement: Text S1 — Detailed description of the models and parameters used. (PDF) [file pone.0028293.s001.pdf]

Supporting Material

miR451 and AMPK mutual antagonism in glioma cell migration and proliferation : A mathematical model

Yangjin Kim, Soyeon Roh, Sean Lawler, Avner Friedman

S1. Detailed description of the models and parameters used.

Parameter estimation

Diffusion coefficients : D_n, D_P, D_G

D_n : For EC cells migrating in a culture containing angiogenic factor, Stokes and Lauffenburger [1] measured the diffusion coefficient to be 7×10^{-9} cm^2/s. A 'typical' animal's cell motility coefficient has been estimated to be 5×10^{-10} cm^2/s [2]. A smaller value, 10^{-10} cm^2/s, was used in [3]. While the experimental results for motility of human glioma [4] and glioblastoma cells [5] in 2D substrate suggest a value of D_n in the range of 1.16×10^{-10}-2.31×10^{-9} cm^2/s, Stein *et al.* [6] used a 10-fold higher value of D_n (=2.31×10^{-8} cm^2/s) in order to get a better fit to the experimental data. Burgess *et al.* [7] took D_n=1.7×10^{-9} cm^2/s, but Sander and Deisboeck [8] argued that D_n should be much smaller, namely, 10^{-12} cm^2/s. We shall take D_n=10^{-11} cm^2/s.

D_P : In experiments of the movement of MMP-1 in the collagen fibril, Saffarian *et al.* [9] estimated the diffusion coefficient to be $(8\pm1.5)\times10^{-9}$ cm^2/s for wild-type activated MMP-1 and $(6.7\pm1.5)\times10^{-9}$ cm^2/s for inactive mutant. In our simulation, we take $D_P = 5 \times 10^{-11}$ cm^2/s.

D_G : The diffusion coefficient of G was measured as 6.7×10^{-7} cm^2/s in the brain [10] and 1.3×10^{-6} cm^2/s in collagen gel [11]. The diffusion coefficient in a growing tumor spheroid or aggregate is much smaller than the one in the medium, and so we take $D_G = 2.31 \times 10^{-7}$ cm^2/s.

chemo/hapto-taxis coefficients : χ_n, χ_n^1

In the presence of EGF, glioma cells travelled a distance 0.4-0.5cm in 150 hrs [12]; glioma cells in agar containing EGF travelled faster, covering a distance of 1.25cm in 150 hrs, while in plain agar they travelled only 0.75cm during the same period. In experiments with U87MGmEGFR spheroid growth, Deisboeck *et al.* [13] calculated the cell velocity to be in the range of $50-110$ $\mu m/24h$. Kim *et al.* [14] assumed that gradient of the glucose concentration was $3\times10^{-3}-10^{-4}$ g/cm^4 and a drift velocity $25-110$ $\mu m/24h$ of mobile cells to compute an intermediate value of $\chi_n = \frac{velocity}{gradient} = 2.76 \times 10^{-4}$ $cm^5/(g.s)$. In this paper, we assume that the chemotactic sensitivity is relatively small due to the fluctuating glucose level. We take χ_n=1.86×10^{-7} $cm^5 g^{-1} s^{-1}$.
For the haptotactic sensitivity, we take $\chi_n^1 = 4.17 \times 10^{-5}$ $cm^5/(g.s)$.

other parameters

In the next subsection we shall determine reference values n^*, ρ^*, P^*, G^* for n, ρ, P, G.

λ_{11} (tumor cell proliferation) : Doubling time were in the range from $27h$ (U87MG) to $60h$ (LN405) for human glioma cells [15]; this translates into proliferation rate of $(7.1-8)\times10^{-6}s^{-1}$. Measured value of proliferation rate were reported as $1/day$, or $8\times10^{-6}s^{-1}$, in typical experiments of Sander and Deisboeck [8]. Taking into consideration that large flux of glucose being supplied periodically in our system, we take λ_{11}=1.112×10^{-4} s^{-1}.

λ_{31} (MMP production) : It is difficult to measure the MMP production rate directly. The range of $(1.11-6.94)\times10^{-8}$ s^{-1} was estimated in [14] for sparse migrating cells. The MMP production rate,

written as $\lambda(n,\rho)$, was modeled by $\lambda_{31}n$ in [14] where $\lambda_{31} = 6.94 \times 10^{-8}\ s^{-1}$. Here, we model it as $\lambda_{31}n\rho$, because ρ is expected to oscillate quite significantly. In order to adjust to the order of magnitude of MMP production in [14], we take an estimated value, $\lambda_{31} = 6.95 \times 10^{-5}\ cm^3g^{-1}s^{-1}$; see also [15].

λ_{32} (decay rate of MMP) : MMP is secreted by a tumor cell and is highly localized (fast decay) in the invading front of migrating tumor cells. We assume half-life of MMP to be very short (approximately 3.8 h) so that $\lambda_{32} = 5.0 \times 10^{-5}\ s^{-1}$.

λ_{41} (consumption rate parameters) : Nutrient consumption rate was measured as $\alpha = 1.6\ pg/cell/min$ in [16]. We compute λ_{41} from $\lambda_{41}G^\dagger = \alpha$ when $G^\dagger = 8.9\times10^{-4}\ g/cm^3$ is between high ($4.5\times10^{-3}\ g/cm^3$) and low ($3.0 \times 10^{-4}\ g/cm^3$). Hence, $\lambda_{41} = \frac{\alpha}{G^\dagger} = 0.3\ cm^3/(g.s)$.

Table 2 summarizes all the above parameter values.

Nondimensionalization

Tumor module

Table 3 lists reference values. We take $T = 1\ hour$ and $L = 1.0\ mm$, so that so that $D = \frac{L^2}{T} = 2.78 \times 10^{-6}\ cm^2/s$. We determine the reference values for n, ρ, P, G as follows:

n^* : We take n^*=$1\times10^{-3}\ g/cm^3$. This is based on Chicoine *et al.* [12] who plated human glioma cells with density 2.5-5$\times10^5$ *cells/ml* in agar gel to investigate the cell migration in the presence of growth factors.

ρ^* : The main ECM component is collagen. Kaufman *et al.* (2005) investigated several patterns of different collagen I concentrations, and estimated ρ to be 0.5-2.0 mg/ml for glioma spheroids of diameter $\sim 200\mu m$. Stein *et al.* (2007) reported on experiments where U87 and U87ΔEGFR were implanted into collagen I of concentration of 2.6 mg/ml. We take ρ^*=$1.0 \times 10^{-3}\ g/cm^3$ as our reference value of ECM as in [14].

P^* : Recently, it was observed that PCK3145 has the ability to downregulate MMP-9 level for prostate cancer patients with high levels of MMP-9 > 100 $\mu g/l$ [17]. We take $P^* = 1.0 \times 10^{-7}\ g/cm^3$ as in [14].

G^* : Sander and Deisboeck (2002) [8] used the characteristic concentration of glucose $2\times10^{-4}\ g/cm^3$, and took the boundary condition $6 \times 10^{-4}\ g/cm^3$ for glucose concentration far from the tumor (see also [13]). In [18], high ($4.5\ g/l = 4.5 \times 10^{-3}\ g/cm^3$) and low ($0.3\ g/l = 3.0 \times 10^{-4}\ g/cm^3$) glucose levels were introduced. We take this high value as a reference value so that dimensionless value of glucose ($G = 1$) corresponds to the high glucose level.

We nondimensionalize the variables and parameters in the partial differential equations (11)-(14) as follows:

$$\bar{t} = \frac{t}{T},\ \bar{x} = \frac{r}{L},\ \bar{n} = \frac{n}{n^*},\ \bar{n}_0 = \frac{n_0}{n^*},\ \bar{G} = \frac{G}{G^*},\ \bar{\rho} = \frac{\rho}{\rho_*},\ \bar{\rho}_0 = \frac{\rho_0}{\rho_*},\ \bar{P} = \frac{P}{P^*},$$
$$\bar{D}_n = \frac{D_n}{D},\ \bar{D}_P = \frac{D_P}{D},\ \bar{D}_G = \frac{D_G}{D},\ \bar{\lambda}_{11} = T\lambda_{11},\quad \bar{\lambda}_{21} = T\lambda_{21}P^*,$$
$$\bar{\lambda}_{22} = \lambda_{22}T,\ \bar{\lambda}_{31} = \frac{\lambda_{31}Tn^*\rho^*}{P^*},\ \bar{\lambda}_{32} = T\lambda_{32},\ \bar{\lambda}_{41} = T\lambda_{41}n^*,\ \bar{\lambda}_{42} = \frac{T\lambda_{42}}{G^*},$$
$$\bar{\chi}_n = \frac{\chi_n G^* T}{L^2},\ \bar{\chi}_n^1 = \frac{\chi_n^1 \rho^* T}{L^2}.$$

We choose λ_G and λ_ρ such that $\bar{\lambda}_G = \bar{\lambda}_\rho = 1$. If we drop the bar ("-") in the new variables and parameters, then the differential equations remain unchanged.

Internal dynamics module

In order to mathematically model the dynamical network shown in Figure 3 the dimensional version of the dynamical system for the internal dynamics was derived as follows:

$$\frac{dm}{dt} = g + \frac{\Lambda_1 \Lambda_2^2}{\Lambda_2^2 + \Lambda_5 a^2} - \mu_1 m,$$
$$\frac{da}{dt} = s + \frac{\Lambda_3 \Lambda_4^2}{\Lambda_4^2 + \Lambda_6 m^2} - \mu_2 a,$$

where $m(t)$ and $a(t)$ are concentrations of miR-451 and the AMPK complex at time t, s is source of the complex, g is the glucose level within the experimental system, Λ_1 and Λ_3 are the autocatalytic enhancement parameters for miR-451 and the complex respectively, Λ_2 and Λ_5 are the Hill-type inhibition saturation parameters from the counter part of miR-451 and the complex respectively, Λ_5 is the inhibition strength of miR-451 by the complex, Λ_6 is the inhibition strength of the complex by miR-451, μ_1 and μ_2 are the decay rates of miR-451 and the complex respectively.

The following dimensionalization was performed to get the dimensionless key control equations in the main section

$$T = \mu_1 t,\ M = \frac{m}{m^*},\ A = \frac{a}{a^*},\ G = \frac{g}{\mu_1 m^*},\ S = \frac{s}{\mu_2 a^*},\ k_1 = \frac{\Lambda_1}{\mu_1 m^*},$$
$$k_2 = \Lambda_2,\ k_3 = \frac{\Lambda_3}{\mu_2 a^*},\ k_4 = \Lambda_4,\ \alpha = \Lambda_5 (a^*)^2,\ \beta = \Lambda_6 (m^*)^2,\ \epsilon = \frac{\mu_1}{\mu_2}.$$

miRs are typically more stable than their targets [19, 20] and the parameter ϵ is small [21]. Typical half-life of AMPK is measured to be 6 h [22] ($\mu_2 \sim 0.12 h^{-1}$) while the half-life of a miRNA is much larger 101-225 h in a recent study [23]. We take a slightly larger half-life of miR-451, 290 h, leading to $\mu_1 = 0.0024\ h^{-1}$ and $\epsilon = \frac{\mu_1}{\mu_2} = 0.02$. miRNA concentrations in an animal cell (assuming 1000-25,000 μm^3 volume) were estimated to be 80 $pM - 2.2\ \mu M$ [24] and we take our reference value $m^* = 1.0\ \mu M$. Based on the high (4.5 g/l) and low (0.3 g/l) glucose level in [18] and m^*, we estimate glucose supply rate through several pathways $g = (2.4 \times 10^{-5} - 2.4 \times 10^{-3})\ \mu M/h$ resulting in a range of dimensionless glucose input levels $G = \frac{g}{\mu_1 m^*} = 0.01 - 1.0$. AMPK concentration was measured as 35-150 nM in rat liver [25] and we take $a^* = 100 nM$. We take the signal source of the AMPK complex, $s = 2.4\ nM/h$ leading to $S = \frac{s}{\mu_2 a^*} = 0.2$. The autocatalytic rate (Λ_1) of miR-451 is assumed to be 4-fold larger than its negative contribution ($\mu_1 m^*$) from its decay in the absence of inhibition pathway from the AMPK module, $k_1 = \frac{\Lambda_1}{\mu_1 m^*} = 4.0$ (Similarly for its counterpart, the AMPK complex, we take $k_3 = \frac{\Lambda_3}{\mu_2 a^*} = 4.0$). The Hill-type dimensionless parameters Λ_2, Λ_4 (and their corresponding k_2, k_4 without change) are fixed to be equal to 1. Finally, the inhibition strength ($\alpha = 1.6$) of miR-451 by the AMPK complex was assumed to be a bit stronger than the inhibition strength ($\beta = 1.0$) of the AMPK complex by miR-451.

Table 1 summarizes all the above parameter values for the internal dynamics model (1)-(2).

References

1. Stokes CL, Lauffenburger DA (1991) Analysis of the roles of microvessel endothelial cell random motility and chemotaxis in angiogenesis. J theor Biol 152: 377–403.

2. Bray D (2000) Cell Movements: From Molecules to Motility. Garland Pub.

3. Anderson ARA, Chaplain MAJ (1998) Continuous and discrete mathematical models of tumor-induced angiogenesis. Bull Math Biol 60: 857–900.

4. Demuth T, Hopf N, Kempski O, Sauner D, Herr M, et al. (2000) Migratory activity of human glioma cell lines in vitro assessed by continuous single cell observation. Clin Exp Metastasis 18: 589-97.

5. Hegedus B, Zach J, Czirok A, Lovey J, Vicsek T (2004) Irradiation and taxol treatment result in non-monotonous, dose-dependent changes in the motility of glioblastoma cells. J Neurooncol 67: 147-57.

6. Stein A, Demuth T, Mobley D, Berens M, Sander L (2007) A mathematical model of glioblastoma tumor spheroid invasion in a three-dimensional in vitro experiment. Biophys J 92: 356-65.

7. Burgess P, Kulesa P, Murray J, EC J (1997) The interaction of growth rates and diffusion coefficients in a three-dimensional mathematical model of gliomas. J Neuropathol Exp Neurol 56: 704-13.

8. Sander L, Deisboeck T (2002) Growth patterns of microscopic brain tumors. Phys Rev E 66: 051901.

9. Saffarian S, Collier I, Marmer B, Elson E, Goldberg G (2004) Interstitial collagenase is a brownian ratchet driven by proteolysis of collagen. Science 306: 108-11.

10. Jain R (1987) Transport of molecules in the tumor interstitium: a review. Cancer Res 47: 3039-51.

11. Rong Z, Cheema U, Vadgama P (2006) Needle enzyme electrode based glucose diffusive transport measurement in a collagen gel and validation of a simulation model. Analyst 131: 816-21.

12. Chicoine M, Madsen C, Silbergeld D (1995) Modification of human glioma locomotion in vitro by cytokines egf, bfgf, pdgfbb, ngf, and tnf alpha. Neurosurgery 36: 1165-70; disbrain cancer.

13. Deisboeck T, Berens M, Kansal A, Torquato S, Stemmer-Rachamimov A, et al. (2001) Pattern of self-organization in tumour systems: complex growth dynamics in a novel brain tumour spheroid model. Cell Prolif 34: 115-134.

14. Kim Y, Lawler S, Nowicki M, Chiocca E, Friedman A (2009) A mathematical model of brain tumor : pattern formation of glioma cells outside the tumor spheroid core. J Theo Biol 260: 359-371.

15. Mercapide J, Cicco R, Castresana J, Klein-Szanto A (2003) Stromelysin-1/matrix metalloproteinase-3 (mmp-3) expression accounts for invasive properties of human astrocytoma cell lines. Int J Cancer 106: 676-82.

16. Li C (1982) The glucose distribution in 9l rat brain multicell tumor spheroids and its effect on cell necrosis. Cancer 50: 2066-73.

17. Annabi B, Bouzeghrane M, Currie J, Hawkins R, Dulude H, et al. (2005) A psp94-derived peptide pck3145 inhibits mmp-9 secretion and triggers cd44 cell surface shedding: implication in tumor metastasis. Clin Exp Metastasis 22: 429-39.

18. Godlewski J, Nowicki M, Bronisz A, Palatini GNJ, Lay MD, et al. (2010) Mircrorna-451 regulates lkb1/ampk signaling and allows adaptation to metabolic stress in glioma cells. Molecular Cell 37: 620–632.

19. Khanin R, Vinciotti V (2008) Computational modeling of post-transcriptional gene regulation by micrornas. J Comput Biol 15: 305-316.

20. Yao G, Lee T, Mori S, Nevins J, You L (2008) A bistable rb-e2f switch underlies the restriction point. Nat Cell Biol 10: 476-482.

21. Aguda B, Kim Y, Hunter M, Friedman A, Marsh C (2008) Microrna regulation of a cancer network: Consequences of the feedback loops involving mir-17-92, e2f, and myc. PNAS 105: 19678-19683.

22. Crute B, Seefeld K, Gamble J, Kemp B, Witters L (1998) Functional domains of the alpha1 catalytic subunit of the amp-activated protein kinase. J Biol Chem 273: 35347-54.

23. Gantier M, McCoy C, Rusinova I, Saulep D, Wang D, et al. (2011) Analysis of microrna turnover in mammalian cells following dicer1 ablation. Nucleic Acids Res 39: 5692-703.

24. Ragan C, amd MA Ragan MZ (2011) Quantitative prediction of mirna-mrna interaction based on equilibrium ' concentrations. PLoS Comput Biol 7: e1001090:doi:10.1371/journal.pcbi.1001090.

25. Hardie D, Salt I, Hawley S, Davies S (1999) Amp-activated protein kinase : an ultrasensitive system for monitoring cellular energy charge. Biochem J 338: 717-722.
